# Highly Sensitive Perovskite Photoplethysmography Sensor for Blood Glucose Sensing Using Machine Learning Techniques

**DOI:** 10.1002/advs.202405681

**Published:** 2024-09-20

**Authors:** Yongjian Zheng, Zhenye Zhan, Qiulan Chen, Jianxin Chen, Jianwen Luo, Juntao Cai, Yang Zhou, Ke Chen, Weiguang Xie

**Affiliations:** ^1^ Siyuan Laboratory, Guangdong Provincial Engineering Technology Research Center of Vacuum Coating Technologies and New Energy Materials, College of Physics & Optoelectronic Engineering Jinan University Guangzhou Guangdong 510632 China; ^2^ Department of Medical Devices Guangdong Food and Drug Vocational College Guangzhou 510520 China; ^3^ Department of Cardiovascular Medicine The First Affiliated Hospital of Jinan University Guangzhou Guangdong 510630 China; ^4^ Guangzhou Research Institute of Optical Mechanical and Electronical Technologies Co.,Ltd Guangzhou Guangdong 510663 China; ^5^ Guangdong Provincial Key Laboratory of Optical Fiber Sensing and Communications Jinan University Guangzhou Guangdong 510632 China

**Keywords:** blood glucose monitoring, machine learning, near‐infrared, perovskite photodetectors, vapor deposition

## Abstract

Accurate non‐invasive monitoring of blood glucose (BG) is a challenging issue in the therapy of diabetes. Here near‐infrared (NIR) photoplethysmography (PPG) sensor based on a vapor‐deposited mixed tin‐lead hybrid perovskite photodetector is developed. The device shows a high detectivity of 5.32 × 10^12^ Jones and a large linear dynamic range (LDR) of 204 dB under NIR light, guaranteeing accurate extraction of eleven features from the PPG signal. By a combination of machine learning, accurate prediction of blood glucose level with mean absolute relative difference (MARD) as small as 2.48% is realized. The self‐powered PPG sensor also works for real‐time outdoor healthcare monitors using sunlight as a light source. The potential for early diabetes diagnoses by the perovskite PPG sensor is demonstrated.

## Graphical abstract

A mixed tin‐lead hybrid perovskite photodetector by vapor deposition method is presented, which shows high detectivity and LDR under the NIR region. The superior photoresponse stability guarantees the repeatable and accurate measurement of the PPG signal with blood glucose information and shows the potential of non‐invasive blood glucose detection in near‐infrared applications.

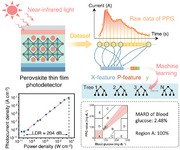

## 1 Introduction

Diabetes poses a serious threat to global health.^[^ ^1^ ^]^ It is estimated that by 2035, there will be 592 million diabetic patients worldwide.^[^ ^2^ ^]^ Blood glucose monitoring is required in the daily treatment of diabetes patients, however, the current invasive techniques, which require pricking of the blood from the fingertips, are distressing for patients. Meanwhile, it has potential risks for trauma and blood infection diseases.

To address this issue, various minimally‐invasive (including Dexcom^[^ ^3^ ^]^ and Medtronic^[^ ^4^, ^5^ ^]^ companies, etc.)^[^ ^6^, ^7^ ^]^ and non‐invasive blood glucose monitoring techniques (such as infrared spectroscopy,^[^ ^8^, ^9^, ^10^, ^11^ ^]^ Ramon spectroscopy,^[^ ^12^ ^]^ electrocardiogram,^[^ ^13^ ^]^ sweat detection,^[^ ^14^ ^]^ enzymatic technology,^[^ ^15^ ^]^ transdermal technology^[^ ^16^ ^]^) have been proposed. Minimally‐invasive techniques offer the advantage of reducing pain and the risk of infection while maintaining high accuracy compared to traditional blood glucose monitoring. However, they are costly and require periodic replacement and calibration. Non‐invasive techniques improve patient compliance in continuous blood glucose measurement. Nevertheless, they are still limited by accuracy, technical complexity, and individual differences.

PPG, a low‐cost and non‐invasively optoelectronic technique that measures the reflected or transmitted light intensity caused by the changes of blood volume in the tissue vascular bed, has been widely used in clinical physiological monitoring of heart rate (HR) as well as arterial blood oxygen saturation (SaO_2_). However, due to the differences between individuals and the performance of the photodetector, accurate non‐invasive blood glucose monitoring using the PPG method is still challenging. PPG signal carrying blood glucose information of patients lies in the NIR wavelength. Detecting the slight change in blood volume requires an NIR photodetector with high NIR sensitivity. Besides, how to extract the features and establish their relationship with individual blood glucose levels is still unclear.^[^ ^17^, ^18^, ^19^, ^20^ ^]^

Benefiting from low production cost, straightforward preparation procedure, and excellent optoelectrical performance, organic–inorganic hybrid perovskite has attracted extensive interest in photodetection. Mixed tin‐lead hybrid perovskites not only have a narrower bandgap than traditional pure lead perovskites, extending the highly efficient responsivity from visible to NIR region but are also less toxic to both humans and the environment. Benefiting from these advantages, mixed tin‐lead hybrid perovskite devices have received significant attention in human health monitoring. Several pioneer works on PPG signal measurement have shown the potential application of perovskite photodetector in pulse and blood oxygen measurement,^[^ ^21^, ^22^, ^23^, ^24^ ^]^ it's then attractive whether the performance of the perovskite photodetector can be improved to improve the accuracy of PPG blood glucose sensor.

In this work, we report a vapor‐deposited mixed tin‐lead hybrid perovskite photodetector as a PPG sensor with high sensitivity under NIR illumination. Eleven different temporal and spectral features were extracted from the PPG signals and mapped to the blood glucose values through machine learning. Eventually, a MARD as small as 2.48% is realized. Meanwhile, the high sensitivity allows the PPG sensor to operate under sunlight without an additional light source.

## 2 Results

**Figure 1:**
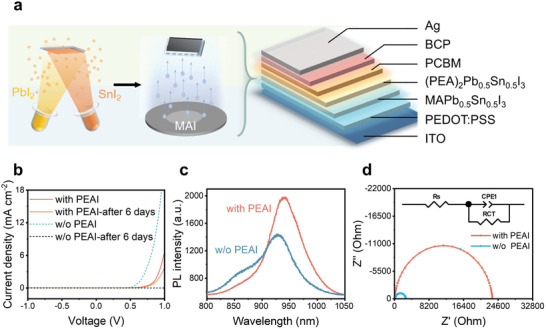
Preparation and optimization of perovskite photodetector. A) The vapor deposition of the perovskite photodetector and the structure of the perovskite photodetector. B) Current density–voltage characteristics of the device with PEAI and without PEAI. The device with PEAI shows only slight attenuation after being placed in the N_2_ atmosphere for 6 days. C) Comparison of steady PL spectra of perovskite thin film with and without PEAI. D) Nyquist plots of devices with and without PEAI at V = 0 V in the dark.

Vapor deposition techniques are widely used in the semiconductor industry which is a toxic‐solvent‐free method for large‐scale fabrication of high‐performance perovskite thin film optoelectrical devices. The thin film fabricated by this vapor deposition is uniform and dense, therefore, it has great potential in preparing large‐area devices and array devices.^[^ ^25^, ^26^, ^27^, ^28^ ^]^ In addition, the combination of perovskite crystallization kinetics and vapor deposition can further improve the perovskite deposition rate and improve the quality of perovskite. Here, the perovskite layer is prepared by sequence vapor deposition as shown in **Figure** **1**a and the entire flow chart is shown in Figure S1 (Supporting Information). The ratio of Pb/Sn in the thin film is controlled by the evaporation rate of 0.5 Å s^−1^. The mix‐thin film is exposed to MAI vapor for the growth of perovskite thin film.^[^ ^29^ ^]^ Compared to PTAA, NiO_x_, Meo‐2PACZ, devices with PEDOT:PSS hole transporting layer show better‐working stability in the dark (Figure S2, Supporting Information).

Large LDR and weak light sensitivity are particularly important to PPG measurement since a slight change of light intensity should be extracted from the strong background. Since Sn^2+^ is easily oxidized to Sn^4+^, there is serious instability under illumination that reduces both the strong and weak light sensitivity, and thus reduces the LDR (Figure S3, Supporting Information). To suppress the effect, both Cs doping and PEA_2_Pb_0.5_Sn_0.5_I_4_ top layer are compared. It's found that molten salt strategy doping Cs needs to be further optimized in both low light intensity and strong light intensity (Figure S4, Supporting Information). By contrast, the PEA_2_Pb_0.5_Sn_0.5_I_4_ top layer has significantly improved LDR by extending the high‐intensity illumination stability and the low‐intensity illumination responsivity. Figure S5 (Supporting Information) shows the AFM images of the perovskite film with or without spin‐coating PEAI. Since the vacuum gas phase method and the vertically oriented growth of perovskite are conducive to the growth of dense and smooth films, the films have a dense morphology with few pinholes. The smooth and dense films are conducive to protecting the film from oxygen penetration, thereby reducing the oxidation of perovskite. Spin‐coated a layer of PEAI on the perovskite, generated a 2D perovskite film surface with large grain sizes and less clear grain boundaries covering the perovskite light‐absorbing layer to isolate external oxygen. Figure 1b shows the significant improvement in device stability after spin‐coating PEAI on the perovskite layer. Although the stability of the device spin‐coated with PEAI has been greatly improved (Figure S6, Supporting Information), it also limits the carrier transmission to a certain extent, resulting in a reduction in photocurrent and photoresponse, which is related to the PEAI concentration (Figure S7, Supporting Information). By adjusting the concentration of the PEAI to adjust the generation of 2D perovskite, finally, 2 mg mL^−1^ PEAI, which is relatively sensitive in weak light, is selected.

Steady photoluminescence (PL) measurements are conducted to verify the stability. Figure 1c shows that both the perovskite film with and without PEAI has an emission peak near 940 nm. However, the thin film without PEAI shows lower emission intensity, indicating higher non‐radiative trap‐assisted recombination loss of carriers. Besides, it also has a peak at ≈860 nm, which represents the defects formed by Sn^2+^ being oxidized to Sn^4+^.^[^ ^30^ ^]^ To further evaluate the defect effect, Nyquist plots of devices at the bias of 0 V in the dark were measured in Figure 1d. The recombination resistance (R_rec_) of 23246 Ω in the device with PEAI is significantly higher than that of the device without PEAI (Rrec = 2587 Ω). This indicates an efficient suppression of charge recombination in the device. Furthermore, the device with PEAI exhibits smaller series resistance and larger recombination resistance, suggesting that the photogenerated carriers in the device are less likely to be captured by defect states, thereby reducing non‐radiative charge recombination.

As a result, the structure of the device was finally configured as indium tin oxide (ITO)/poly(3,4‐ethylenedioxythiophene) polystyrene sulfonate (PEDOT:PSS)/CH_3_NH_3_Pb_0.5_Sn_0.5_I_3_ (MAPb_0.5_Sn_0.5_I_3_) /PEA_2_Pb_0.5_Sn_0.5_I_4_/[6,6]‐phenyl‐C_61_‐butyric acid methyl ester (PC_61_BM)/2,9‐dimethyl‐4,7‐diphenyl‐1,10‐phenanthroline (BCP) /silver (Ag), depicted in Figure 1a.

**Figure 2:**
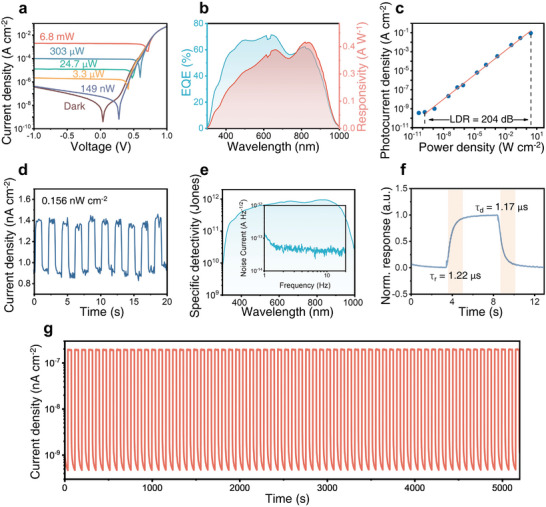
Performance of perovskite photodetector. A) Current density–voltage characteristics. B) External quantum efficiency (EQE) and spectra responsivity (SR) (A W^−1^). C) Photocurrent curves under varied light intensities, LDR reaches 204 dB. D) Photocurrent on‐off switching properties of 0.156 nW cm^−2^. E) Specific detectivity of the perovskite photodetector at 1Hz and the insert shows the noise current spectrum. F) Photo‐transient response of the device under an optical modulation frequency of 100 kHz. G) Continuous tracking of photoresponse stability during a period of 5200 s under modulated illumination intensity of 0.22 µW cm^−2^. The above device is encapsulated and working under zero bias (self‐powered mode) in air under the illumination of 860 nm if not mentioned.

**Figure** **2**a shows the IV characteristic of the optimized photodetector using a PEAI concentration of 2mg mL^−1^. Figure 2b shows that the external quittance efficiency (EQE) is over 60% and a peak responsivity of 0.41 A W^−1^ lies at 860 nm. Figure 2c shows an LDR as large as 204 dB. Figure 2d shows that under illumination as weak as 0.156 nW cm^−2^, the device still exhibits a clear photocurrent of 434 pA cm^−2^. Figure 2e measured the dark current noise spectrum of the photodetector. The human heartbeat frequency lies at ≈1–3 Hz. The noise current is from 1.96 × 10^−13^ A Hz^−1/2^ to 3.4 × 10^−14^ A Hz^−1/2^, corresponding to the specific detectivity (D*) from 1.0 × 10^12^ cm Hz^1/2^ W^−1^ (Jones) to 5.32 × 10^12^ Jones (Figure S8, Supporting Information). The rise time of photoresponse is defined as the time required to increase from 10% to 90% of maximum photocurrent, and the decay time is the time required to decrease from 90 to 10%. Figure 2f shows the self‐powered perovskite photodetector achieved a fast rise time of 1.22 µs, and a fall time of 1.17 µs, which meets the application requirements of a blood glucose monitor. Figure 2g shows that under air conditions, the device shows a uniform and stable photoresponse in 5200 s. The superior photoresponse stability guarantees the repeatable and accurate measurement of the PPG signal. Table S1 (Supporting Information) summarizes the state‐of‐the‐art perovskite IR photodetectors. It shows that the performance of the vapor‐deposited perovskite IR photodetector is among the best.

**Figure 3:**
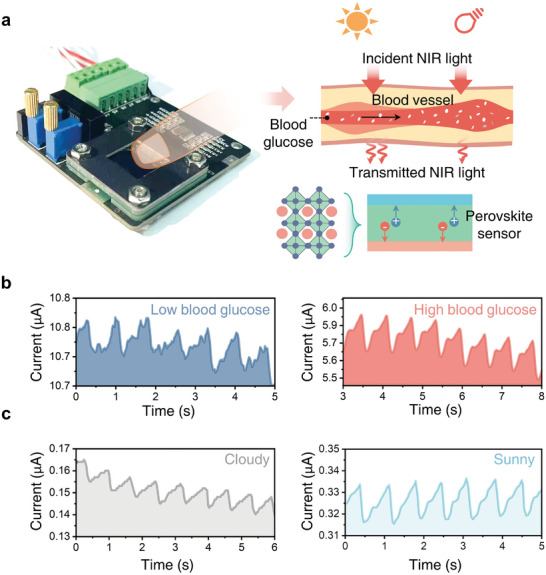
Measurement of PPG signals. A) Schematic of PPG signal measurement and the developed PPG sensor. The device is powered under both indoor and outdoor illumination, and the complete PPG signals can be detected under the light with an NIR band. B) PPG signals under different concentrations of blood glucose. C) PPG signals under different weather.

**Figure** **3** shows our measurement setup for PPG signal measurement. The light intensity penetrating the fingertip is measured using the encapsulated perovskite photodetector. The change of light intensity is determined by the blood volume in the blood vessels that changes all the time with the blood circulatory system (Figure 3a). Glucose shows absorption peaks centered at 850 nm, 940 nm, and 1240 nm, which are distinguishable from blood^[^ ^31^, ^32^ ^]^ (Figure S9, Supporting Information). The change of transmitted or reflected light intensity of these wavelengths is normally used for measurement.^[^ ^8^ ^]^ The PPG signal is measured using transmitted mode with an illumination wavelength of 860 nm, because this wavelength can penetrate the finger (Figure S9, Supporting Information), and it corresponds to the peak responsivity of the photodetector and is close to the absorption peaks of glucose at 850 nm. Figure 3b shows the PPG signals at the high blood glucose level after eating and the low blood glucose level before eating. When the blood glucose level is high, blood glucose plays a major role in the absorption of light intensity, and the feature information of PPG signals is obvious. When the blood glucose level is low, the light through the fingertip is less affected by the blood glucose, so the PPG signals are noisy. Due to the excellent weak light sensitivity, it's found that even without the illumination of a laser, the NIR component of the sunlight is also able to produce obvious and repeatable PPG signals. Figure 3c and Figure S10 (Supporting Information) show that under outdoor conditions, no matter sunny or cloudy, obvious and repeatable PPG signals can be detected.

**Figure 4:**
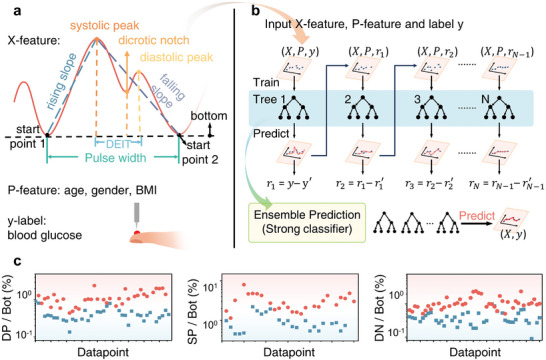
Feature extraction from the PPG signal and training for blood glucose measurement. A) Definition of 11 PPG features as X‐feature, personal information of patient as P‐feature, and corresponding reference blood glucose value y as the label. Each PPG feature is extracted separately from the PPG signal. B) Principle of gradient boosting decision tree regressor which has the best prediction result in our work. C) Three typical PPG features that show a strong correlation with blood glucose. The red point is measured under high blood glucose, and the blue one is under low blood glucose (systolic peak (SP), diastolic peak (DP), dicrotic notch (DN), bottom (Bot)).

Before direct measurement of the blood glucose, a machine learning model should be trained, whose flowchart is shown in Figure S11 (Supporting Information). To extract PPG features, the PPG signal is first measured over a period of time. The initial lowest point of the first double‐peak PPG signal is chosen as the starting point. Based on the starting point, the feature information as shown in **Figure** **4**a can be extracted. The same features of the subsequent PPG signals are extracted in the same way. Eleven PPG features are defined to extract the relative change of glucose absorption due to the change in blood volume so that the relationship between the glucose concentration and the PPG signal can be established. The mean values of each PPG feature (X‐feature), personal information of the patient (P‐feature), and the measured blood glucose using a traditional blood sensor by finger pricking (y‐label) as shown in Table S2 (Supporting Information) are used as input to the machine learning model. As shown in Figure 4b, there will be a first perdition of blood glucose value y’ and an error r_1_ to the reference value y. The *r*_1_, X‐feature, and P‐feature are input in the next training cycle, and *r_N_* is generated in the N cycle.

The idea of the gradient boosting decision tree regressor (GBDT) is a model in the form of an ensemble of multiple weak learners, which combines the predictions of all the weak learners built with a given learning algorithm to improve the generality/robustness of a single estimator. To improve the reliability of the model, multiple data collected from different individuals as shown in Table S3 (Supporting Information) are used to train the model.

The correlation between PPG features and blood glucose is shown in Figure S12 (Supporting Information). Figure 4c shows the three typical feature values of $\frac{\mathrm{systolic}\;\mathrm{peak}}{\mathrm{bottem}}$, $\frac{\mathrm{diastroic}\;\mathrm{peak}}{\mathrm{bottem}}$, $\frac{\;\mathrm{dicrotic}\;\mathrm{notch}}{\mathrm{bottem}}$, which have a high correlation with blood glucose. It illustrates that for different people, high blood glucose and low blood glucose generate significantly different PPG feature information. This proves the reliability of blood glucose measurement using these PPG features.

To find out the most suitable model for measurement of blood glucose, 10 different machine learning and convolutional neural network models, including convolutional neural networks (CNN), support vector machine (SVM), k‐nearest neighbors regressor (KNN), ridge regression (RR), AdaBoost regressor (AdaBoost), linear regression (LR), extra tree regressor (ETR), bagging regressor (BGR), decision tree regressor (DTR), random forest regressor (RFR), gradient boosting decision tree regressor (GBDT) are trained. The metrics of the model for measurement will be evaluated by the mean square error (MSE) loss, root mean squared error (RMSE), mean absolute error (MAE), MARD, and R^2^ score defined in the model evaluation in the supplementary information. For MSE, RMSE, MAE, and MARD, the smaller the better, and for the R^2^ score, the closer to one the better.

**Figure 5:**
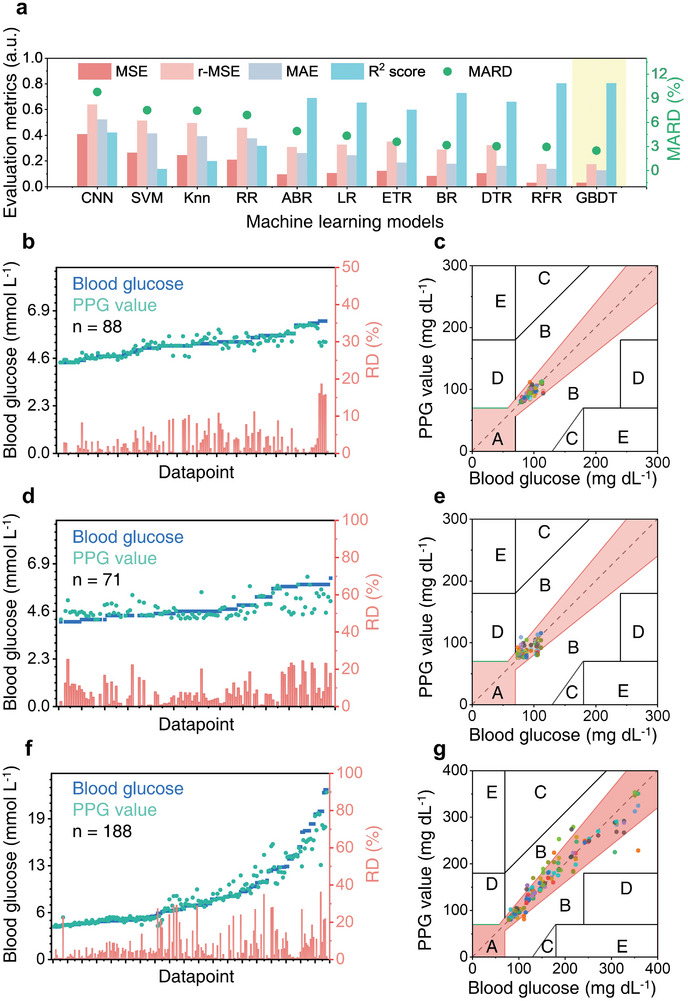
Measurement by PPG sensor. A) Comparison of measurement accuracy using different trained models. B) and C) Comparison and the Clarke Error Grid between the values from the blood glucose meter and the PPG measured values using an 860 nm laser. D) and E) Comparison and the Clarke Error Grid between the blood glucose and the PPG measured values using a solar simulator. F) and G) Comparison and the Clarke Error Grid between the blood glucose and the PPG measured values of different diabetic patients using an 860 nm laser. (n is the sample size of the testing set).

The comparison of 10 models for real measurements is illustrated in **Figure** **5**a and Table S4 (Supporting Information), encompassing 88 data points from a single individual under various conditions, with blood glucose values ranging from 4.4 mmol L^−1^ to 6.4 mmol L^−1^. Detailed data is plotted in Figure 5b, accompanied by the relative error (RD) of each data point. It was observed that both Random Forest Regression (RFR) and Gradient Boosting Decision Trees (GBDT) demonstrated superior performance in terms of Mean Squared Error (MSE), Root Mean Squared Error (RMSE), Mean Absolute Error (MAE), and R‐squared (R2) scores. GBDT further exhibited the lowest MARD at 2.48%. Additionally, **Table** **1** showcases current blood glucose prediction results from different groups, with our PPG sensor delivering the most accurate results. Consequently, we opted for GBDT for our forthcoming measurements.

**Table 1:** Performance of PPG blood glucose sensors from different groups.

Group	Hina^[^ ^9^ ^]^	Hammourr^[^ ^16^ ^]^	Joshi^[^ ^24^ ^]^	Liao^[^ ^35^ ^]^	Alghlayini^[^ ^36^ ^]^	Our work		
Year	2020	2023	2020	2023	2023	2024		
Technology	940 nm SVR fine	880 nm 6 models^a)^	940 nm 1300 nm iGLU 2.0	940 nm LRCN	660nm, 880nm CNN	860 nm GBDT	Sunlight GBDT	860 nm GBDT
MARD (%)	7.62	12.8	4.86	–	–	2.48	7.78	6.93
RMSE (mmol L^−1^)	0.62	0.98	0.75	0.62	0.95	0.17	0.53	2.38
Number of features	6	18	–	–	–	11	11	16
Amount of data^b)^	200	244	1024	–	190	292	237	49 Patients 626
Percent in Clarke A region	>95%	90%	100%	99% in A and B	92.85%	100%	90.7%	93%

^a)^ The models employed include linear regression, support vector machines, Gaussian process regression, kernel approximation models, ensemble models, and neural networks (NNs);

^b)^ Data including both training and measurement.

Despite the small MARD, Figure 5b illustrates deviations of some measured values from the actual values. To assess the clinical applicability of a blood glucose sensor, the Clarke error grid, introduced in 1987 (see Note S4 Supporting Information),^[^ ^33^ ^]^ was used. The data from Figure 5b is plotted on the Clarke error grid shown in Figure 5c, with the x‐axis representing the glucose value from the blood glucose meter and the y‐axis representing the measured result from our PPG sensor. All data points in this study fall within region A on the grid, indicating that they could lead to clinically correct decisions. This demonstrates the potential of our perovskite PPG sensor for non‐invasive blood glucose monitoring. Real‐time blood glucose measurements using our perovskite blood glucose sensor were demonstrated in Movie S1 (Supporting Information) with an LED intensity of 5 mW, showing sensitive and rapid measurements within a few seconds.

The perovskite photodetector exhibits low light sensitivity, as illustrated in Figure 3c. This prompts the investigation of whether the infrared (IR) light in sunlight could serve as a viable light source for blood glucose measurement. To explore this, a standard solar simulator is employed as the light source. Following the training process, the PPG sensor, using the GBDT model, produces 71 measured results depicted in Figure 5d. These results showcase the best performance, with a MARD of 7.78% (Table S5, Supporting Information). Furthermore, Figure 5e demonstrates that 90.7% of the measured blood glucose values are situated within region A, with the remainder in region B. This highlights the potential of the perovskite PPG sensor for real‐time outdoor healthcare monitoring using sunlight as a light source.

In order to assess the performance of the perovskite PPG sensor on different individuals, further tests were carried out on 49 volunteers and some of them with type‐II diabetes. Figure 5f presents a comparison of 188 measured results, revealing that certain data points, particularly those near high blood glucose levels, exhibited a larger deviation from the actual value. This was largely due to the limited mobility of the patients, resulting in unstable and weak PPG signals. However, the MARD remained at 6.93%. The data in the Clarke Error Grid in Figure 5g indicated that 93% of the measured blood glucose values fell within region A, with the remainder in region B, which is deemed acceptable for early clinical diagnosis. Based on the findings of this experiment, a strategy for future applications involving training and a calibration process can be established. Initially, the model will be trained using extensive data from diverse patients to ensure an approximate accuracy of ≈10% for any individual. Subsequently, when applied to a single person, the model will require personal information, the initial PPG measurements, and their corresponding blood glucose levels to calibrate the model for that individual. This approach could yield a personalized model with improved accuracy better than 3%. A comparison of this strategy with recent invasive blood glucose measurement methods and minimally‐invasive devices is presented in **Table** **2**. It can be found that the perovskite PPG sensor is comparable to the state‐of‐art blood glucose sensor. During the measurement of different patients, we found that the alignment of the optical path and the finger caused significant changes in the PPG signal (shown in Figure S13, Supporting Information). This may be because the veins in the fingers are relatively sparse.^[^ ^34^ ^]^ Here, fixing the optical path and using a clamp to fix the fingers is used to avoid errors. At the same time, a finger mold to ensure the standardization of the measurement is required in the future. Second, the absorption of different people's skin is different. Especially PPG signals from those elder people are smaller than the young, the influence can be reduced to a certain extent by adding the P‐feature. This also requires further accumulation of sufficient data from different types of people and building specific models. Thirdly, more complex algorithms may be designed to meet the requirement of PPG signal processing, which could help to further improve the accuracy. The PPG sensor takes advantage of its small size and cost‐efficiency. With improved accuracy, it is a promising non‐invasive continuous blood glucose monitoring technique for patients.

**Table 2:** Comparison of different blood glucose measurement techniques developed in recent years.

Technique	Method	Position	Data number	MARD [%]	RMSE [mmol L^−1^]	Percent in Clarke A region	Year Ref.
Cyclic voltammetry with sweat	–	–	a healthy male and female	–	–	100%	2022^[^ ^37^ ^]^
Cyclic voltammetry with tear	Regression function	In eye	20 participants	–	–	100%	2024^[^ ^38^ ^]^
Electrochemical impedance spectroscopy with Sweat	Decision tree	–	3 participants	–	0.15	–	2022^[^ ^39^ ^]^
Wired Enzyme™ Technology	–	Arm Abdomen	12,677 data	17.3	–	98% in A and B	2003^[^ ^40^ ^]^
Electrochemical Microneedle/ Dexcom G7	–	–	–	–	8.2	–	2024^[^ ^41^ ^]^
Electrochemical Microneedle/ MiniMed 770G	–	–	–	–	8.7‐11	–	2024^[^ ^41^ ^]^
Electrocardiogram	SFF‐WCIM	Back	21 participants	13.42	1.49	99.49 in A and B	2023^[^ ^12^ ^]^
Raman spectroscopy	Bagging‐ABC‐ELM	Blood sample	106 data	–	0.19	100%	2023^[^ ^42^ ^]^
Raman spectroscopy	Partial least squares regression	Hand	160 data	14.8	1.6	99.8% in A and B	2023^[^ ^43^ ^]^
NIR technology	FFNN	Finger	575	1.94	0.71	100%^a)^	2024^[^ ^44^ ^]^
NIR technology	niGLUC‐2.0v	Finger & Wrist	101	–	0.47 (finger) 0.077 (wrist)	100%	2024^[^ ^45^ ^]^
PPG	GBDT	Finger	Individual 292 data	2.48	0.17	100%	This work
			Individual 237 data	7.78	0.53	90.7% in A and 9.3% in B	
			49 Patients 626 data	6.93	2.38	93% in A 7% in B	

^a)^ Surveillance error grid.

## 3 Discussion

NIR perovskite PPG sensor was fabricated by vapor deposition. A 2D perovskite via PEAI on the 3D perovskite layer greatly improved the stability and performance of the device. The device shows a high detectivity of 5.32 × 10^12^ Jones and a high LDR of 204 dB under the NIR region. The excellent weak light intensity ensured accurate measurement of characters by blood glucose changes in the PPG signal. Based on the extraction of features, different models by matching learning are established to improve the measurement accuracy of blood glucose values to 2.48%. Further testing using sunlight and on different people also shows high accuracy which is acceptable in early medical diagnosis. The results show the potential of the perovskite PPG sensor in blood glucose monitor. The green vapor deposition method also provides potential in industrialization and commercialization in the future.

## 4 Experimental Section

### Ethical Approval

All procedures performed in studies involving human participants were in accordance with the ethical standard of the institutional and/or national research committee and with the 1964 Helsinki Declaration and its later amendments or comparable ethical standards.

Appropriate ethics committee approval and informed written consent from all human research participants were obtained.

### Materials and Methods

PEDOT:PSS (CLEVIOS™ P VP AI 4083) solution, PEAI, and BCP were purchased from Xi'an POLED. PbI_2_ (99.999%) was purchased from Sigma–Aldrich. SnI_2_ (99.999%) was purchased from AIYAN. MAI (99.8%) was purchased from TCI. PCBM was purchased from Advanced Election Technology CO, Ltd. All of the chemicals were used as received.

### Device Fabrication

The devices were prepared on cleaned ITO substrates. PEDOT:PSS solution (filtered through a 0.22 µm filter) is spin‐coated onto the ITO substrates as the HTL at 4,000 rpm. for 30 s and baked at 150 °C for 10 min. 200 nm‐thick PbI_2_ and SnI_2_ were deposited onto the PEDOT:PSS surface using dual‐source thermal evaporation and they all have a deposition rate of 0.5 Å s^−1^. The as‐prepared PbI_2_ and SnI_2_ films were then placed face down on a 2 cm height corundum boat with 200 mg MAI powder uniformly dispersed at the bottom. The corundum boat was then placed into a vacuum oven preheated to 180°C, and then pumped down to 10 kPa in no more than 10 min. MAI was sublimed onto the PbI_2_‐SnI_2_ layer and reacted in situ to form perovskite thin films. Then PEAI (in isopropanol, 2 mg mL^−1^) was spin‐coated on the perovskite film at 1,000 rpm. for 30 s and baked at 100 °C for 5 min. The PCBM (in chlorobenzene, 20 mg mL^−1^) and BCP (in isopropanol, 1 mg mL^−1^) were spin‐coated on the perovskite film at 1,000 rpm. for 30 s and at 4,000 rpm. for 30 s after PEAI, respectively. Finally, the silver electrode was thermally evaporated through a shadow mask to complete the device. And the device area of 0.1 cm^2^ was determined by the overlap of the ITO and the metal electrodes. For the PPG sensor in the measurement setup, the perovskite thin film was encapsulated with glue and glass as thick as 0.7 mm. It prevents the direct contact of the finger of the patients to the perovskite thin film, and the release of lead to the environment, which ensures measurement safety.

### Characterizations—Thin Film Characterization

Surface morphology was acquired using an NT‐MDT Ntegra Prima atomic force microscope under ambient conditions with semicontact mode. X‐ray diffraction (XRD) spectra were acquired using a Rigaku MiniFlex 600. Steady‐state photoluminescence (PL) spectra measurements were taken with a 532 nm laser source at room temperature (Horiba, LabRAM HR Evolution Inc.).

### Device Characterization

The devices were characterized without encapsulation (if not mentioned) under the atmosphere and at ambient temperature. During the measurement, the device was posited in a dark metal box to avoid interference from ambient light. Current–voltage characteristics were measured using Keithley 2612A source meters. The external quantum efficiency (EQE) spectrums were acquired under a series of bias voltages using the Enlitech QE‐R system. Incident light power intensity was calibrated by an optical power meter (Daheng Optics, GCI‐080302) and attenuated by a series of absorptive neutral density filters (Thorlabs). The noise current measurements were performed at room temperature and in dark conditions by using a lock‐in amplifier (Sine Scientific Instruments, OE2031). The response time was acquired by an oscilloscope (Tektronix, TDS2024C) with input resistances of 50 Ω. In the final setup, PPG was measured NI USB‐6003 data acquisition device and an 860 nm Laser (DPSSL DRIVER II).

### Statistical Analysis

During the data preprocessing process, python was used to process the csv file saved the PPG waveform, and the PPG waveform of the analog signal was transformed into a discrete characteristic digital signal.

To evaluate the uncertainty of a measurement, aleatoric uncertainty and epistemic uncertainty were suggested in the guide to the expression of measurement (GUM). However, there was no unified calculation method in the machine learning model. In this study, the variance of model predictions estimates aleatoric uncertainty. Epistemic uncertainty was estimated by using ensemble methods. A detail of the evaluation of the uncertainty is given in the Uncertainty Estimation section in Note S3 (Supporting Information).

There were 49 volunteers for this work. The sample size (n) of NIR blood glucose, solar blood glucose, and multi‐person blood glucose monitoring were 292, 237, and 626. Estimation of the uncertainty by calculating the aleatoric uncertainty and epistemic uncertainty. The variance of model predictions estimates aleatoric uncertainty. The aleatoric uncertainty of the data is 0.03±0.11 mmol L^−1^. Epistemic uncertainty is estimated by using ensemble methods, which is 0.5 mmol L^−1^. And the details are summarized in Tables 1 and 2, and the figure legends of Figure 5 and Figure S14 (Supporting Information). Estimation of the uncertainty following the method in Note S3 Supporting Information. Monte Carlo cross‐validation was used in the cross‐validation strategy, the average MARD was 9.63%, with a minimum of 6.77% and a maximum of 12.2%.

MARD and Clark Error Grid were used to compare the predicted blood glucose with the true blood glucose.

## Conflict of Interest

The authors declare no conflict of interest.

## Author Contributions

Y.Z. and Z.Z. contribute equally to this work. Y.Z., Z.Z., and W.X. conceived and designed the experimental work. Y.Z. and Z.Z. fabricated the perovskite photodetector. Y.Z. preprocessed the dataset and calculated the blood glucose machine learning model. Q.C., J.C., and J.C. provided the technical support. Y.Z. and J.L. carried out the blood glucose measurement in the hospital. Y.Z. and K.C. carry out the materials characterization. W.X., Y.Z. and Z.Z. contributed to the writing of the manuscript and to the analysis of the experimental results. W.X. supervised the project.

## Supporting information



Supporting Information

Supplemental Movie 1

## Data Availability

The data that support the findings of this study are available from the corresponding author upon reasonable request.
