# Supplementary material for: Highly Sensitive Perovskite Photoplethysmography Sensor for Blood Glucose Sensing Using Machine Learning Techniques
Source: Adv Sci (Weinh). 2024 Sep 20;11(43):2405681. doi: 10.1002/advs.202405681 (PMC11578317; doi:10.1002/advs.202405681)
Supplement: Supplementary file 1 — Supporting Information [file ADVS-11-2405681-s002.pdf]

ADVANCED SCIENCE
Open Access

Supporting Information

for *Adv. Sci.*, DOI 10.1002/advs.202405681

Highly Sensitive Perovskite Photoplethysmography Sensor for Blood Glucose Sensing Using Machine Learning Techniques

*Yongjian Zheng, Zhenye Zhan, Qiulan Chen, Jianxin Chen, Jianwen Luo, Juntao Cai, Yang Zhou, Ke Chen and Weiguang Xie**

SUPPLEMENTARY INFORMATION

Highly sensitive perovskite photoplethysmography sensor for blood glucose sensing using machine learning techniques

Yongjian Zheng[1+], Zhenye Zhan[1+], Qiulan Chen[2], Jianxin Chen[3], Jianwen Luo[1], Juntao Cai[4], Yang Zhou[1], Ke Chen[1], Weiguang Xie[1,5*]

[1]Siyuan Laboratory, Guangdong Provincial Engineering Technology Research Center of Vacuum Coating Technologies and New Energy Materials, College of Physics & Optoelectronic Engineering, Jinan University, Guangzhou, Guangdong 510632, China

[2]Department of Medical Devices, Guangdong Food and Drug Vocational College, Guangzhou 510520, China

[3]Department of Cardiovascular Medicine, The First Affiliated Hospital of Jinan University, Guangzhou，Guangdong 510630, China

[4]Guangzhou Research Institute of Optical, Mechanical and Electronical Technologies Co.,Ltd, Guangzhou, Guangdong 510663, China

[5]Guangdong Provincial Key Laboratory of Optical Fiber Sensing and Communications, Jinan University, Guangzhou, Guangdong 510632, China

*Corresponding author. Corresponding authors. E-mail addresses: wgxie@email.jnu.edu.cn (W. Xie).

†These authors contribute equally to this work.

Note S1 - Preparation and optimization of perovskite thin film photodetector

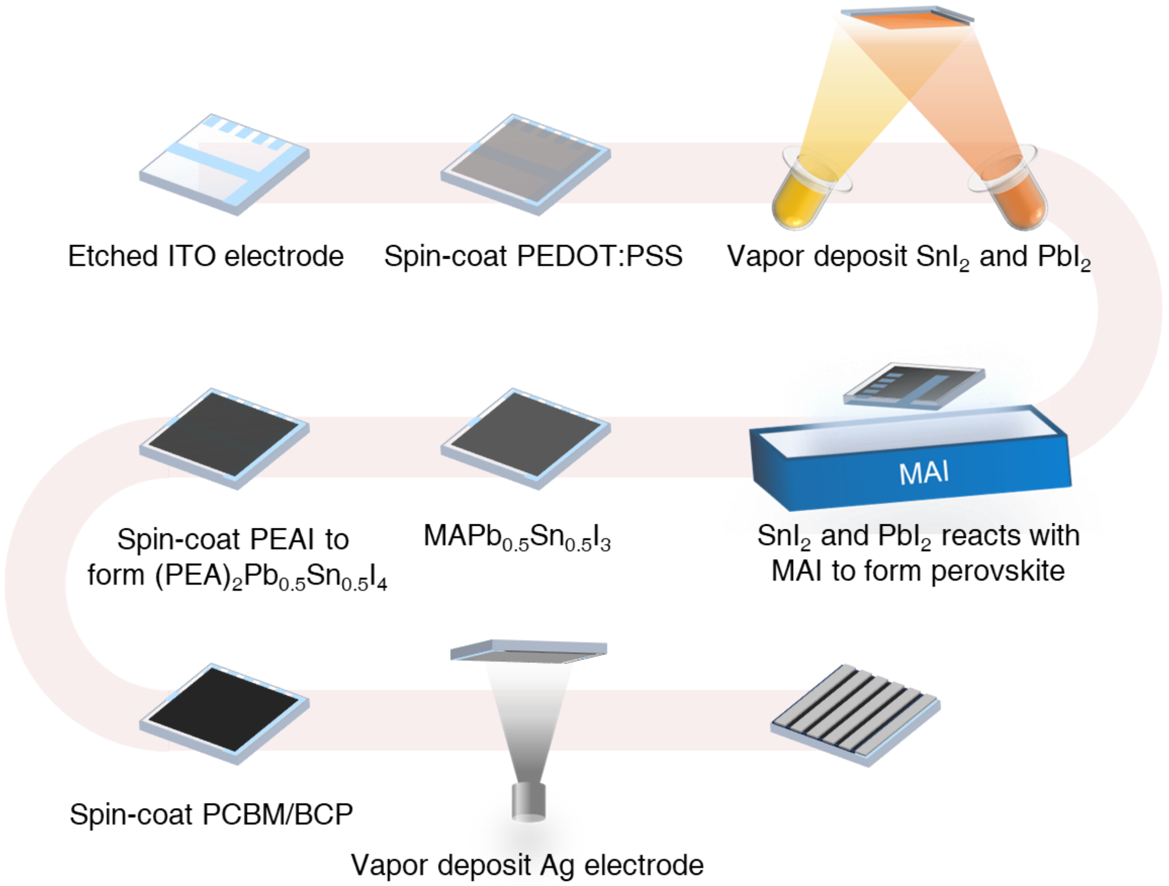

Figure S1. The flow chart of perovskite device preparation.

The process involves co-evaporating a three-dimensional perovskite light-absorbing layer using a two-step method, followed by the generation of a protective layer of two-dimensional perovskite through spin-coating PEAI. The device's structure is configured as indium tin oxide (ITO)/poly(3,4-ethylenedioxythiophene) polystyrene sulfonate (PEDOT:PSS)/$CH_3NH_3Pb_{0.5}Sn_{0.5}I_3$ ($MAPb_{0.5}Sn_{0.5}I_3$) /$(PEA)_2Pb_{0.5}Sn_{0.5}I_4$/[6,6]-phenyl-C61-butyric acid methyl ester (PC61BM)/2,9-dimethyl-4,7-diphenyl-1,10-phenanthroline(BCP)/silver(Ag).

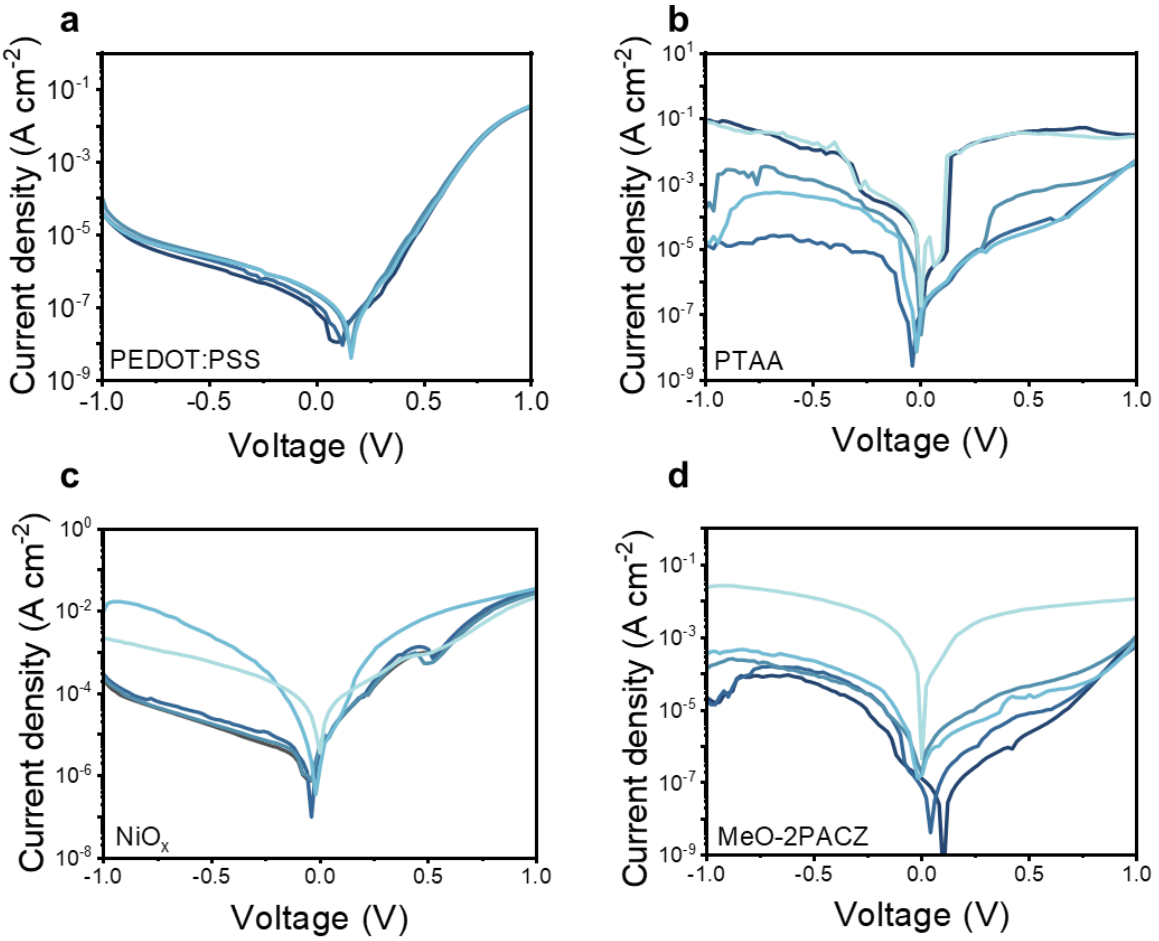

Figure S2. Comparison of different hole transport layers in perovskite photodetectors. The figure shows the dark currents of different electrodes on the same device. a) PEDOT:PSS, b) PTAA, c) NiO_x and d) Meo-2PACZ.

Current density–voltage characteristics of different hole transport show that devices using PEDOT:PSS as the hole transport layer have the best photoresponse and uniformity, and the dark currents of different electrodes on the same device are similar. This also ensures the reproducibility of the device for commercialization.

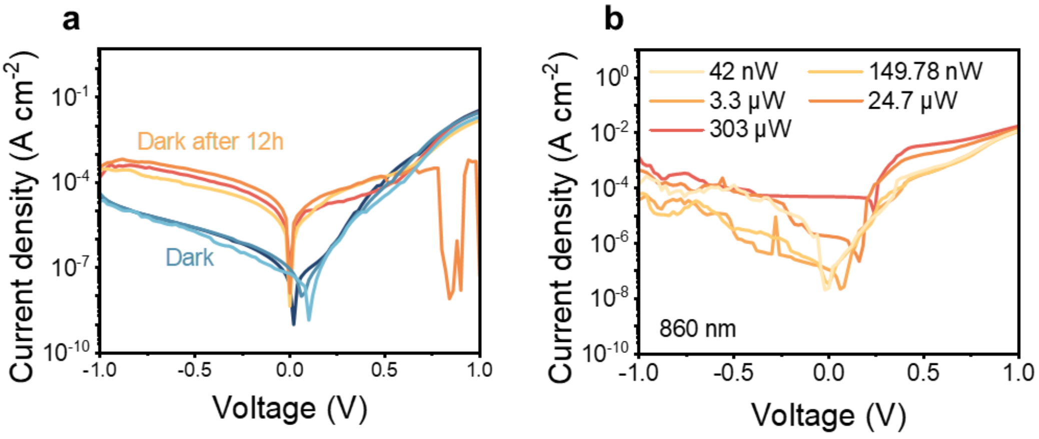

Figure S3. The Characterization of perovskite photodetectors which use PEDOT:PSS as the hole transport layer. a) Dark current J-V characteristics comparison after 12 hours, which shows poor stability. b) Current density–voltage characteristics. The illumination was a NIR light of 860 nm, with a series of intensities. Under illumination, the light current of the device is unstable under negative bias voltage and the device is easily damaged.

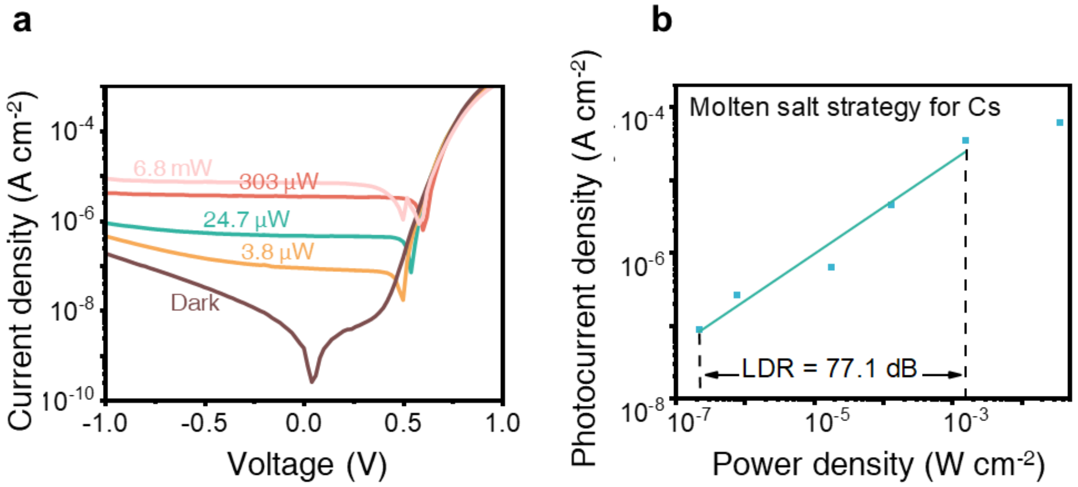

Figure S4. The Characterization of perovskite photodetectors by molten salt strategy. a) Current density–voltage characteristics. b) Photocurrent curves under varied light intensities.

The above device is working in the air under the illumination of 532 nm with a series of intensities from 42 nW to 6.8 mW. At the light intensity of 6.8 mW, the increase of the device's photocurrent is limited, and the

current density-voltage curve starts to be unstable, so the device needs to be further optimized in both low light intensity and strong light intensity. `

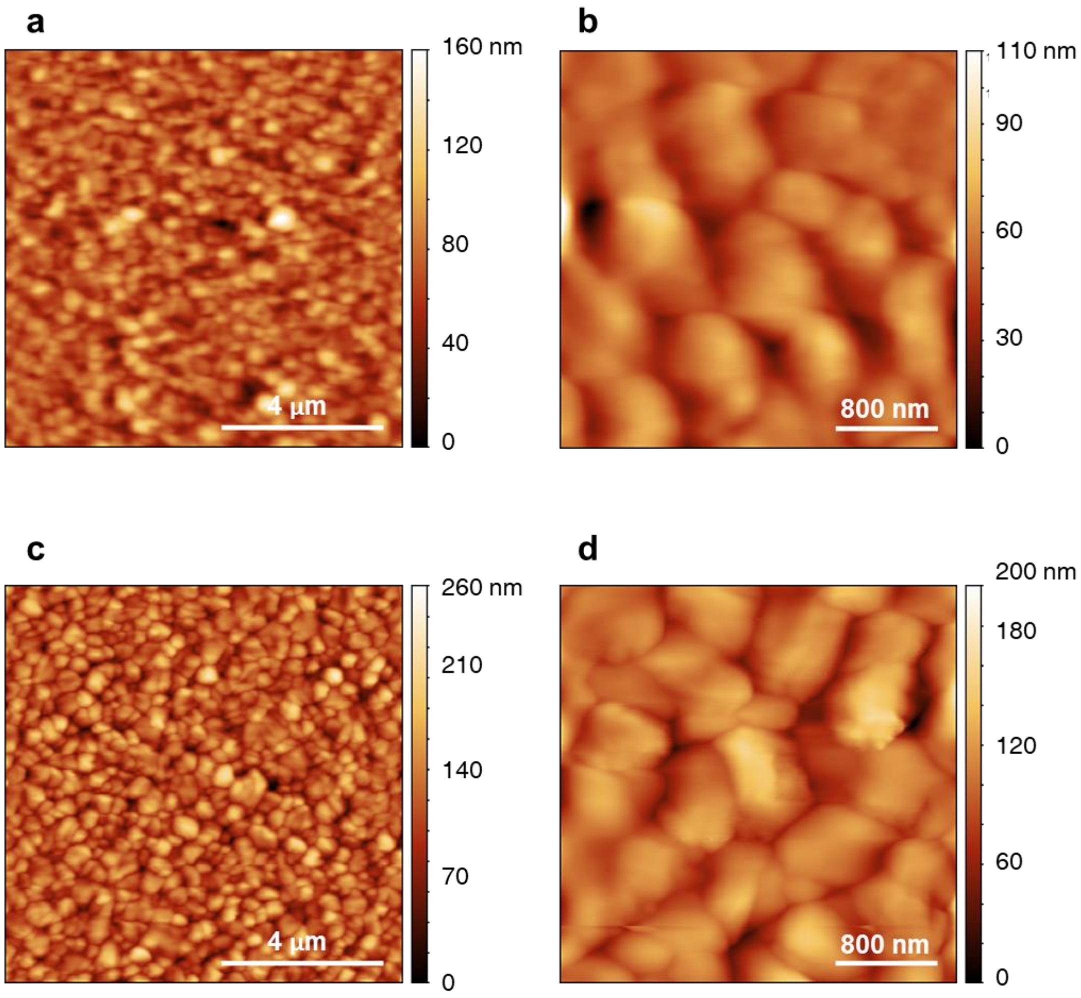

Figure S5. AFM images of the surface morphology. a) and b) with PEAI. c) and d) without PEAI.

Spin-coated a layer of PEAI on the perovskite, generated a two-dimensional perovskite film surface with large grain sizes and less clear grain boundaries covering the perovskite light-absorbing layer to isolate external oxygen.

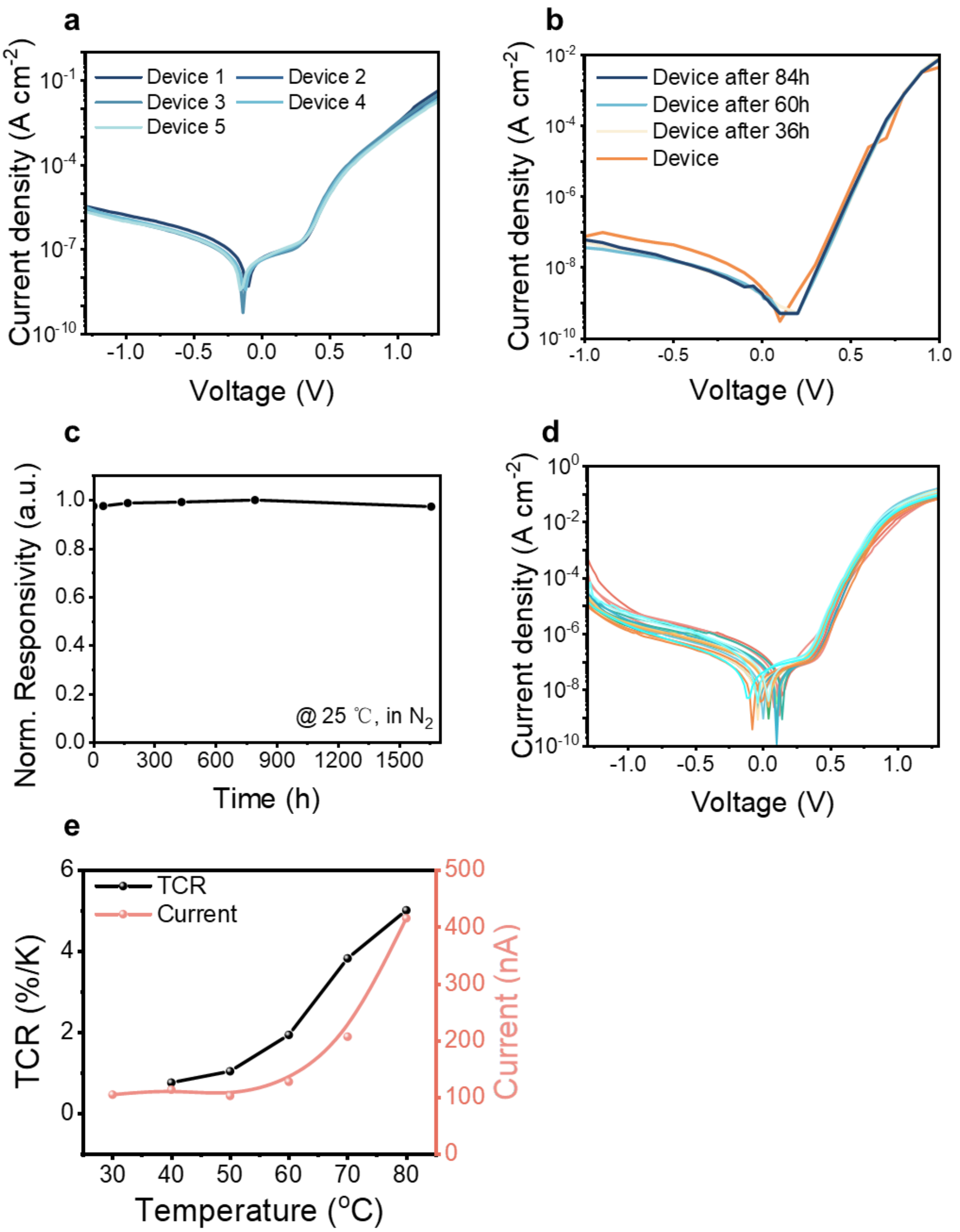

Figure S6. The Characterization of perovskite photodetectors with PEAI. a) The J-V characteristics of five different devices on the same chip in the dark. b) Dark current comparison after 36, 60, and 84 hours, which shows that the stability has been improved. c) The storage stability of the device. d) The J-V characteristics of different devices on the different substrates in the dark. e) Temperature dependence of the device performance.

After optimization by PEAI, the J-V characteristics of devices in the same chip overlap with each other in (a).

It proves the uniformity of thin film prepared by vapor deposition. The device storage stability has been significantly enhanced. The dark J-V characteristics in (b) are almost unchanged after 84 hours. The photoresponse of the device keeps stable for 1600 hours in (c). While the stability is enhanced, the dark J–V characteristics of the device are also similar under different batches, and the uniformity and repeatability of the film are guaranteed.

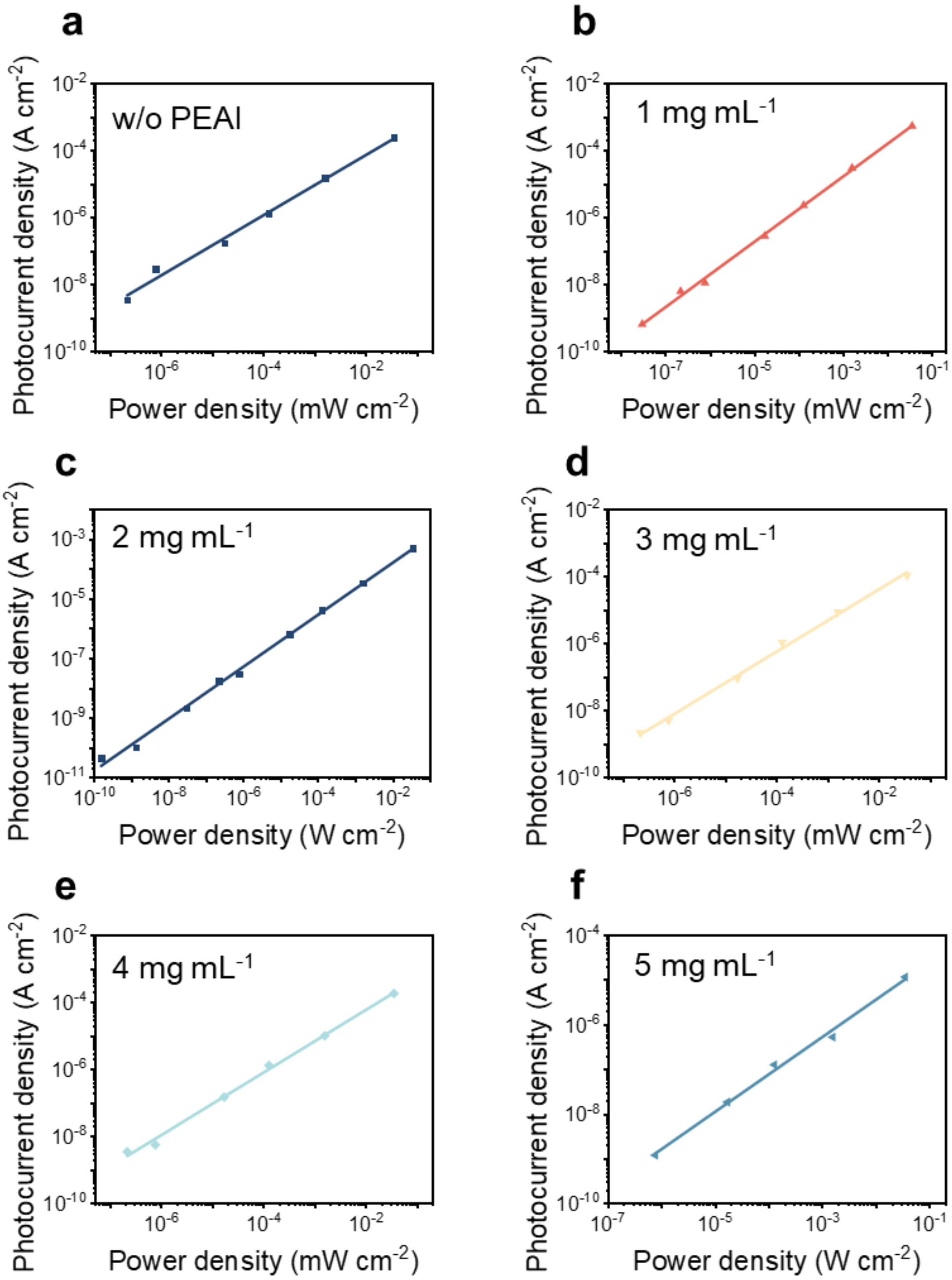

Figure S7. Comparison of LDR in different concentrations of PEAI. a) Without PEAI, b) 1 mg mL^{-1}, c) 2 mg

mL^{-1}, d) 3 mg mL^{-1}, e) 4 mg mL^{-1}, f) 5 mg mL^{-1} PEAI.

After spin-coating PEAI, the LDR of the device is improved. As the concentration reaches 2mg, the weak light response reaches the maximum and then gradually decreases.

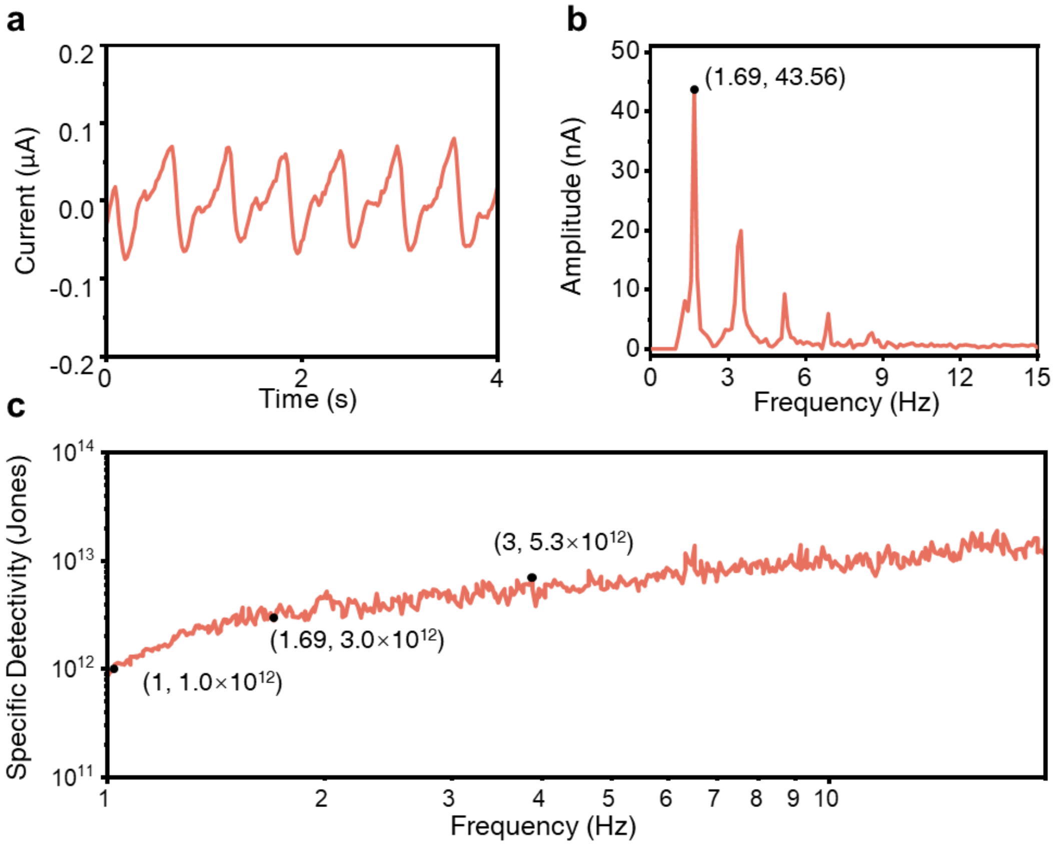

Figure S8. a) PPG signal with FFT filter. b) FFT spectrogram of the PPG signal. The frequency of heartbeat (PPG signal) is 1.69 Hz. c) Specific Detectivity of different frequencies. The illumination was a NIR light of 860 nm.

The human heartbeat frequency lies at around 1 to 3 Hz. The noise current is from 1.96×10^{-13} A Hz$^{-1/2}$ to 3.4×10^{-14} A Hz$^{-1/2}$, corresponding to the specific detectivity (D*) from 1.0×10^{12} cm Hz$^{1/2}$ W^{-1} (Jones) to 5.32×10^{12} Jones. (a) and (b) shows the 101.4 heart rate of a normal human. Our devices work well with low

specific detectivity in the human heart rate range.

Table S1. Comparison with cutting-edge perovskite IR photodetector.

Method	Material & Structure	Spectral Range (nm)	I_d (A cm^{-2})	R (A W^{-1})	D* (Jones)	Rise/fall time	LDR (dB)	Stability	Ref
Solution	ITO/NiO/$MAPbI_3$/PDPP3T:$PC_{71}BM$/LiF/Al	300-910	8.58×10^{-10}	-	1.59×10^{13} (848 nm)	109/49 μs	100	>20 days	[1]
	$MAPbBr_3$ nanoplate	300-800	1.8×10^{-15} (A)	5.04 (2V, 520 nm)	5.37×10^{12} (2V, 520 nm)	80/110 μs	122	-	[2]
	ITO/PTAA/Perovskite/TBA-Azo/C_{60}/BCP/Cu	300-1000	9.61×10^{-8} (-0.1 V)	0.45 (770 nm)	2.21×10^{11} (785 nm)	42.9 ns	185	>300 h	[3]
	ITO/PTAA/Perovskite/IEICO/C_{60}/BCP/Cu	300-900	2.6×10^{-8} (-0.1 V)	0.14 (820 nm)	7.37×10^{11} (820 nm)	27 ns	192	-	[4]
	ITO/SnO_2/$Cs_{0.15}FA_{0.85}PbI_3$/Organic BHJ/Phen-NADPO/Ag	400-1000	~ 2×10^{-11} (-0.4 V)	0.102 (900 nm)	1.6×10^{12} (-1.5 V, 900 nm)	32.3/34.2 μs	>120	-	[5]
	ITO/PTAA/$MAPbI_3$/BHJ/C_{60}/BCP/Cu	400–1000	3.4×10^{-8} (-0.1 V)	0.37 (870 nm)	2.3×10^{11} (870 nm)	5.6 ns	191	-	[6]
	ITO/PTAA/Perovskite (with ATFBA)/C_{60}/BCP/Cu	300-1000	6×10^{-9}	0.52 (850 nm)	5.34×10^{12} (850 nm)	-	224	>450 days	[7]
Vapor	ITO/PEDOT:PSS/$MASn_{0.5}Pb_{0.5}I_3$/$PEA_2Pb_{0.5}Sn_{0.5}I_4$/PCBM/BCP/Ag	300-1000	3.56×10^{-10}	0.41 (860 nm)	5.32×10^{12} (860 nm)	1.22/1.17 μs	204	>1600 h	Our work

* If not mentioned, the data is measured at 0V.

Note S2 - PPG signal extraction and analysis

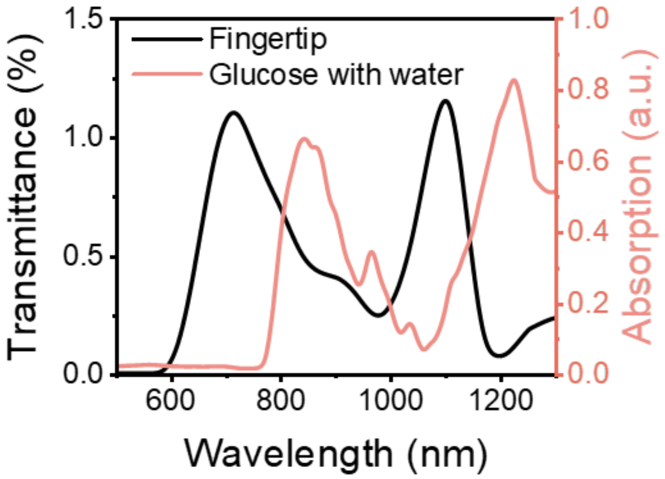

Figure S9. The transmissivity spectrum of fingertip, and absorption spectrum of glucose.

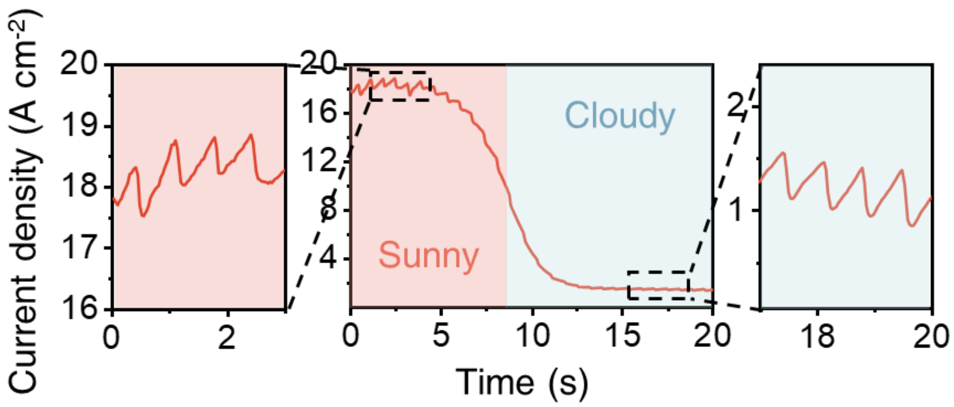

Figure S10. The PPG signals in different weather conditions.

During the process of detecting the PPG signals, the weather conditions changed from sunny to cloudy. The PPG signals can still be detected despite significant changes in light intensity.

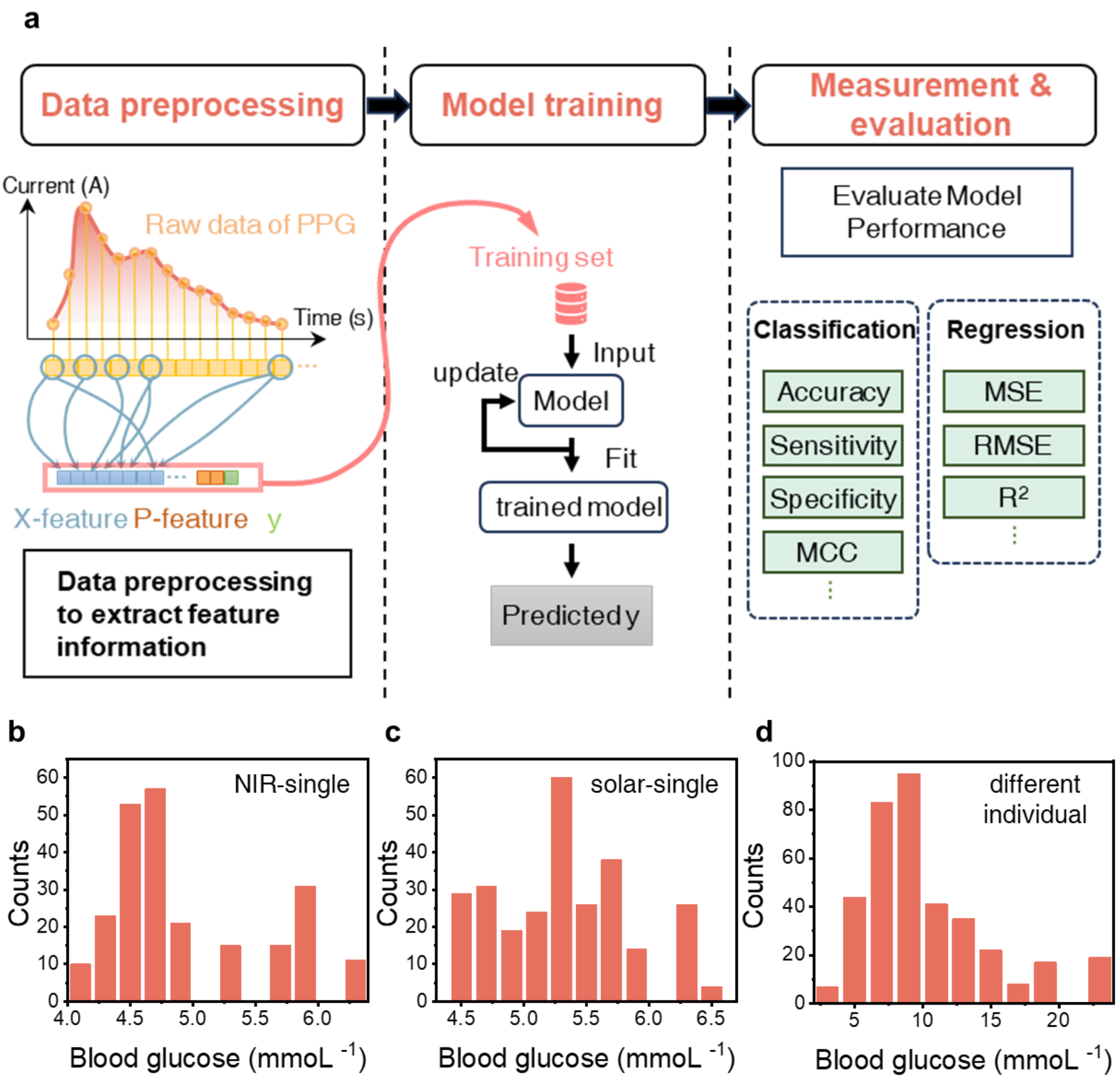

Figure S11. Machine learning to predict blood glucose. a) Flowchart of model training. b) Blood glucose distribution of the dataset of one single person using 860 nm illumination. c) Blood glucose distribution of the dataset of one single person using a solar simulator. d) Blood glucose distribution of the dataset of different individuals using 860 nm illumination.

Figure S11 shows the entire process of training the machine learning model for measurement.

First is the data pre-processing.

Table S2. Dataset for the training of the model

**X-feature:**					
systolic peak	diastolic peak	dicrotic notch	falling slope	rising slope	bottom
systolic peak ratio	diastolic peak ratio	dicrotic notch ratio	pulse width	DEIT	
**P-feature:**					
age	BMI	gender			
**y-label:**					
blood glucose					

Table S2 shows detailed parameters for the model. It contains the features from the PPG signal (X-feature), personal information of the patient (P-feature), and the measured blood glucose using a traditional blood sensor by finger pricking (y-label).

Table S3. The information of Asian volunteers.

	**Age**	**Gender**	**BMI = weight/(height^2) (kg m^{-2})**
Volunteer 1	20	Woman	18.59
Volunteer 2	23	Man	22.16
Volunteer 3	23	Woman	16.82
Volunteer 4	23	Man	20.05
Volunteer 5	23	Man	26.00
Volunteer 6	23	Woman	22.43
Volunteer 7	24	Man	21.05
Volunteer 8	24	Man	21.38
Volunteer 9	24	Man	23.67
Volunteer 10	25	Man	19.13
Volunteer 11	26	Man	22.14
Volunteer 12	26	Man	20.76
Volunteer 13	36	Woman	18.40
Volunteer 14*	40	Man	22.20
Volunteer 15*	42	Woman	21.78
Volunteer 16*	44	Man	24.38
Volunteer 17*	44	Man	41.50
Volunteer 18*	56	Woman	21.53
Volunteer 19*	58	Man	26.24
Volunteer 20*	59	Man	23.85
Volunteer 21*	63	Woman	23.18
Volunteer 22	63	Woman	21.97
Volunteer 23*	65	Woman	21.40
Volunteer 24*	66	Man	24.63
Volunteer 25	66	Man	20.90
Volunteer 26*	67	Man	22.90
Volunteer 27	68	Woman	21.78
Volunteer 28*	69	Man	24.60
Volunteer 29*	70	Woman	27.34
Volunteer 30*	70	Man	24.39
Volunteer 31*	71	Man	23.50
Volunteer 32*	72	Man	23.51
Volunteer 33*	72	Man	23.50
Volunteer 34*	73	Man	29.80
Volunteer 35*	73	Woman	29.80
Volunteer 36	73	Woman	22.00

Volunteer 37*	73	Woman	26.70
Volunteer 38*	73	Man	26.60
Volunteer 39*	74	Woman	27.82
Volunteer 40	75	Woman	20.50
Volunteer 41*	75	Man	24.88
Volunteer 42*	76	Man	24.63
Volunteer 43	77	Woman	21.78
Volunteer 44*	77	Woman	24.27
Volunteer 45	79	Woman	18.20
Volunteer 46*	79	Woman	24.27
Volunteer 47*	82	Man	26.30
Volunteer 48*	85	Woman	21.78
Volunteer 49*	90	Woman	24.65

* The volunteers with type II diabetes.

Second is the model training process.

To train the model for a single volunteer person using laser with 860 nm wavelength, 292 sets of data from a volunteer were collected. The distribution of the blood glucose is shown in **Figure S11**b.

To train the model using a solar simulator,237 sets of data from a volunteer were collected. The distribution of the blood glucose is shown in **Figure S11**c.

To train the model for different individuals, 626 sets of data from 49 volunteers were collected. The distribution of the blood glucose is shown in **Figure S11**d, and the information of the volunteer is given in **Table S3**.

Third, the model is ready for direct measurement.

After training, the above model is used to directly measure the same group of volunteers to test the accuracy of our sensor. The following parameters are used to evaluate the model accuracy.

Mean square error (MSE) loss is also called quadratic loss and L2 loss, and is often used in regression prediction tasks. The mean square error function measures the quality of a model by calculating the square of the distance (i.e., the error) between the predicted value and the actual value:

$$MSE = \frac{\sum\left(y_{pred} - y_{true}\right)^2}{n} \quad (1)$$

That is, the closer the predicted value is to the true value, the smaller the mean square error between the two

is.

Root mean squared error (RMSE), root mean square error (i.e., MSE root). A measurement of the deviation between observed values and true values. It is often used as a standard for measuring the prediction results of machine learning models.

$$RMSE = \sqrt{\frac{\sum\left(y_{pred} - y_{true}\right)^2}{n}} \tag{2}$$

Mean absolute error (MAE), also known as L1 loss function, is a loss function used in regression models. MAE is the sum of the absolute differences between the target variable and the predictor variable:

$$MAE = \frac{\sum\left|y_{pred} - y_{true}\right|}{n} \tag{3}$$

so it measures the average error size in a set of predicted values, regardless of their direction, ranging from 0 ~ ∞.

R^2 score is also called the coefficient of determination or the goodness of fit, which reflects the degree to which the independent variable x explains the changes in the dependent variable y. The closer it is to 1, the better the model fits.

$$R^2\ score = 1 - \frac{\sum\left(y_{pred} - y_{true}\right)^2}{\sum\left(y_{pred} - \bar{y}\right)^2} \tag{4}$$

Mean absolute relative difference (MARD) is the core indicator for evaluating the accuracy of CGM (continuous glucose monitoring) products. Currently, most internationally, MARD less than 15% is used as the standard value for CGM listing. The smaller the value, the closer the blood glucose reading is to the reference value, that is, the higher the accuracy of blood glucose measurement.

$$MARD = \frac{\sum \frac{\left|y_{pred} - y_{true}\right|}{y_{true}}}{n} \times 100\% \tag{5}$$

Relative difference (RD) is also used to evaluate the accuracy of each data to the true blood glucose.

$$RD = \frac{\left|y_{pred} - y_{true}\right|}{y_{true}} \times 100\% \tag{6}$$

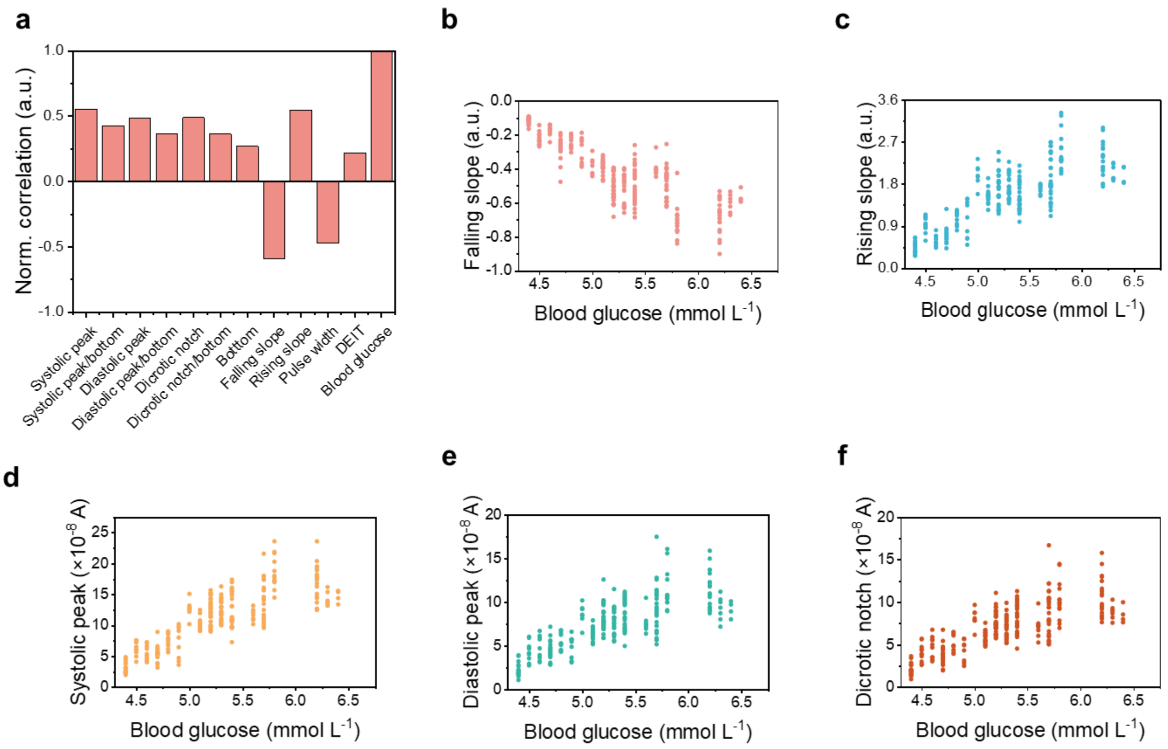

Figure S12. a) Correlation between blood glucose and PPG features. The change of b) Falling slope, c) Rising slope, d) Systolic peak, e) Diastolic peak, f) Dicrotic notch with blood glucose.

Table S4. Accuracy of blood glucose measurement using NIR laser with different methods in this work. Detail about the method can be found in the reference.

NIR Model	MSE (mmol L^{-1})	RMSE (mmol L^{-1})	MAE (mmol L^{-1})	R^2 score	MARD (%)
Convolutional Neural Networks[8]	0.41	0.64	0.52	0.42	9.79
Support Vector Machine[9]	0.26	0.51	0.41	0.14	7.52
K-Nearest Neighbors Regressor[10]	0.25	0.50	0.39	0.20	7.44
Ridge Regression[11]	0.21	0.46	0.38	0.32	6.91
AdaBoost Regressor[12]	0.10	0.31	0.26	0.69	4.91
Linear Regression[13]	0.11	0.33	0.24	0.65	4.33
Extra Tree Regressor[14]	0.12	0.35	0.19	0.60	3.58
Bagging Regressor[15]	0.08	0.29	0.18	0.73	3.16
Decision Tree Regressor[16]	0.10	0.32	0.16	0.66	3.02
Random Forest Regressor[17]	0.03	0.17	0.14	0.80	2.94
Gradient Boosting Decision Tree Regressor[18]	0.03	0.17	0.13	0.81	2.48

Table S5. Accuracy of blood glucose measurement using solar simulator with different methods in this work

Solar Model	MSE (mmol L^{-1})	RMSE (mmol L^{-1})	MAE (mmol L^{-1})	R^2 score	MARD (%)
Ridge Regression	0.33	0.58	0.48	0.08	9.93
Convolutional Neural Networks	0.51	0.71	0.44	-0.40	9.28
Linear Regression	0.26	0.51	0.45	0.27	9.18
K-Nearest Neighbors Regressor	0.30	0.55	0.43	0.17	9.02
AdaBoost Regressor	0.28	0.53	0.43	0.23	8.94
Extra Tree Regressor	0.43	0.65	0.43	-0.18	8.71
Support Vector Machine	0.29	0.54	0.42	0.19	8.54
Random Forest Regressor	0.30	0.54	0.40	0.18	8.31
Bagging Regressor	0.29	0.54	0.40	0.19	8.26
Gradient Boosting Decision Tree Regressor	0.28	0.53	0.37	0.22	7.78

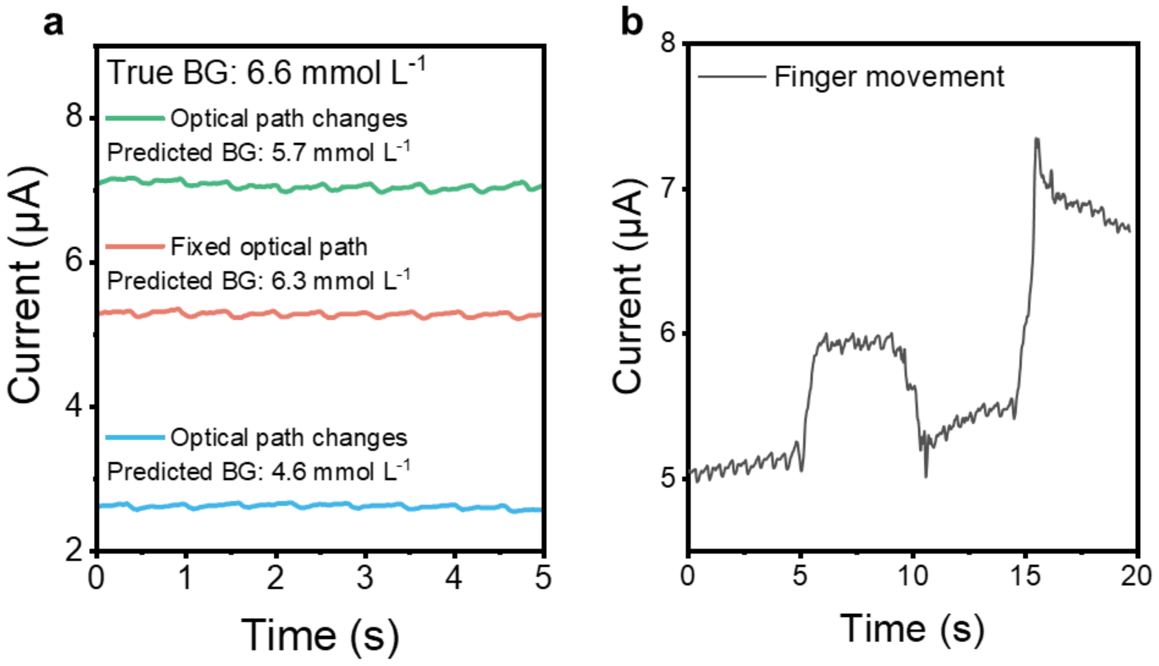

Figure S13 (a) The influence of optical path changes on Blood glucose prediction. (b) The influence of Finger movement on PPG signals detection.

To quantify the influences of the optical path and finger movement, the predicted blood glucose of fixed optical path and optical path changed and the PPG signals of finger movement are tested. **Figure S13**a shows that when the optical path is fixed in the right position, the MARD of predicted blood glucose is 4.5%, and when the optical path is changed, the predicted blood glucose becomes inaccurate. The significant variation of PPG signals can be seen in **Figure S13**b when the finger has subtle movement.

Note S3 - Algorithm transparency

Algorithm:

The algorithm of our work is gradient boosting decision tree regression (GBDT). Gradient Boosting Decision Trees (GBDT) is a machine learning technique for regression and classification tasks. It builds an ensemble of decision trees sequentially, where each subsequent tree corrects the errors made by the previous one. Here's a detailed explanation:

Design:

1. GBDT utilizes the decision trees as the based learners. Decision trees are hierarchical models where the dataset is split into subsets based on the values of input features, and each subset is recursively split until a stopping criterion is met (e.g., maximum is reached).

2. GBDT employs boosting, a technique which each subsequent model in the ensemble focuses on reducing the errors (residuals) of the combined ensemble of models that preceded it. It sequentially adds new models to correct the residuals left by the existing set of models.

3. The 'gradient' in GBDT refers to the gradient descent optimization algorithm used to minimize the loss when adding new models. It calculated the gradient of loss function with respect to the output of the previous model in the ensemble, and used this gradient to fit the new model.

4. GBDT combines multiple weak learners (individual decision trees) to create a strong learner. Each tree is trained sequentially, where each new tree corrects the errors made by the combined ensemble of all previous trees.

In short, GBDT is made up of a series of decision tree models. The initial decision tree model provides a basic prediction, which is not precise. Consequently, the second decision tree model in the sequence attempts to

predict the pseudo-residuals—the difference between the model's predictions and the actual values. The sum of the predictions of the two models still contains pseudo-residuals when compared to the true values, necessitating the involvement of a third model to forecast the pseudo-residuals. This cycle repeats, adding further decision tree models to refine the predictions, until the error between the predictions of all decision tree models and the actual values falls below a specified threshold, signaling the completion of the training process.

Training Process:

1. Initialize the ensemble with a basic prediction often the mean or median of the target variable.
2. For a fixed number of iterations:
    a) Computer the negative gradient of the loss function with respect to the current ensemble's predictions; these gradients represent the pseudo-residuals.
    b) Train a new decision tree on these pseudo-residuals using the training data.
    c) The tree is typically grown to a limited depth and complexity to maintain the weak learner property.
    d) Update the ensemble prediction by adding the scaled prediction of the new tree (multiplied by the learning rate).
    e) Optionally, apply shrinkage (a discount factor) to the tree's predictions before adding them to the ensemble.

3 The process repeats until the maximum number of trees is reached or the improvement in loss is below a certain threshold.

It operates by combining weak learners, typically small decision trees, to form a strong predictive model.

Parameter Selection Process:

We used Grid search to select parameters (including n_estimators, learning_rate, loss, min_sample_split, alpha, max_depth). Grid search is a method used for selecting model parameters during the model training process. It works by defining a grid of parameters that are to be evaluated. Each point on this grid represents a combination of parameter values to be tested. The grid search algorithm then systematically tests each combination using cross-validation to determine the optimal parameters for the model. After identifying the optimal parameter combination using a parameter grid search, we defined a finer, more detailed parameter space within a small range around this combination to further refine the parameters and determine the final parameters.

Cross-validation Strategy:

In the cross-validation strategy, we used Monte Carlo cross-validation, which is a technique used to validate the performance of a machine learning model by repeatedly sampling subsets of the dataset for training and testing. Unlike traditional cross-validation methods where the dataset is split into fixed folds, Monte Carlo cross-validation randomly partitions the data multiple times into training and testing sets. Each partitioning is independent of the others, and the model is trained and evaluated for each partition. We randomly partitioned the dataset a hundred times and trained models separately. The average MARD was 9.63%, with a minimum of 6.77% and a maximum of 12.2%.

Statistical Analysis

The GUM Guidelines offer a methodology for assessing and expressing measurement uncertainty, which quantifies the difference between a measurement result and the true value. This process ensures the reliability and comparability of measurement results. However, GUM mainly focuses on error analysis of experimental measurements, while uncertainty management in machine learning involves a wider range of model design

and data processing techniques. To ensure the accuracy of blood glucose monitoring, we have calculated the aleatoric uncertainty and epistemic uncertainty of our model [19] (illustrated in **Figure S14**), emphasizing the identification and quantification of all sources of uncertainty in the measurement process, similar to GUM. Furthermore, this statistical method, like GUM, provides methods for quantifying uncertainty.

First, aleatoric uncertainty is estimated by the variance of model predictions. Usually, standard deviation is computed using training data because we want to assess the model's performance during training and the stability of its predictions. Training data forms the basis for model fitting, and comparing actual values with predicted values provides an intuitive sense of model error. **Figure S14**a shows that the calculated the mean residual error between the predicted value and the train value is 0.03 mmol L^{-1}, and the standard deviation of the residual error is 0.11 mmol L^{-1}.

Second, we use ensemble methods to compute epistemic uncertainty. By assigning different random initial parameters to the same model, we compute 100 versions of the model with different random initial parameters, and have them predict on the same data. Compute the standard deviation of the prediction helps estimate the epistemic uncertainty. **Figure S14**b shows that the standard deviation of our model’s predicted values is primarily within 0.5 mmol L^{-1}, indicating the strong consistency in predictions across different initial parameters of the GBDT model for the same test data points. Moreover, data points with lower blood glucose levels exhibit stronger consistency.

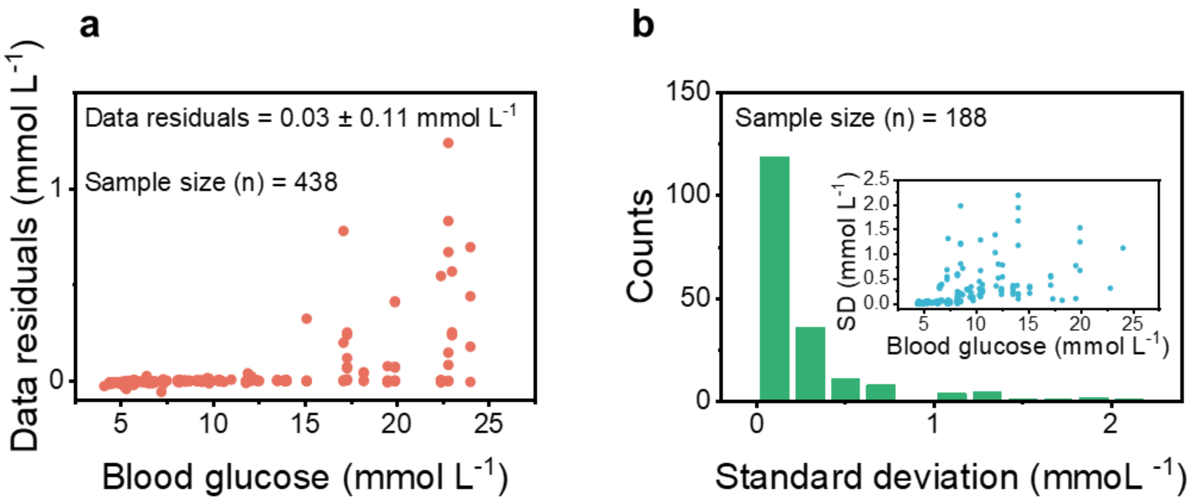

Figure S14 (a) Aleatoric uncertainty of the data. (b) Epistemic uncertainty of the model.

Note S4 – Clinical evaluation using Clarke Error Grid

Clarke Error Grid in **Figure 5**c was introduced by five experts of the University of Virginia in 1987, which provides an intuitive and effective method for the evaluation of blood glucose monitoring systems and helps physicians and patients understand the accuracy of the system and its impact on clinical decision-making[22].

Region A is the region on both sides of the diagonal, the shape of the region results from the judgment that a difference between blood glucose meter values and reference blood glucose values of less than 20% or blood glucose values as well as reference values are in the hypoglycaemic range (< 70mg/dl). The data in region A can help doctors to make clinically correct decisions. In region B, the difference between the blood glucose meter and the reference value is greater than 20%, and the resulting clinical decision-making is not as accurate as region A, but it is still able to provide support for doctors to make correct diagnoses. In both region C and region D, doctors run the risk of making a mistake diagnoses. Data in both E region would cause doctors to make the exact opposite diagnoses, which may endanger the safety of the patients. Table S6 compares the different blood glucose measurement techniques developed in recent years. The PPG sensor shows advantages in convenience and accuracy.

References

[1] Li G, Wang Y, Huang L, et al. High-performance UV–Vis–NIR photodetectors based on perovskite/PDPP3T polymer composites[J]. Ceramics International, 2023, 49(9): 13860-13871.

[2] Mei L, Zhang K, Cui N, et al. Ultraviolet‐Visible‐Short‐Wavelength Infrared Broadband and Fast‐Response Photodetectors Enabled by Individual Monocrystalline Perovskite Nanoplate[J]. Small, 2023: 2301386.

[3] Ma N, Jiang J, Zhao Y, et al. Stable and sensitive tin-lead perovskite photodetectors enabled by azobenzene derivative for near-infrared acousto-optic conversion communications[J]. Nano Energy, 2021, 86: 106113.

[4] Li C, Lu J, Zhao Y, et al. Highly sensitive, fast response perovskite photodetectors demonstrated in weak light detection circuit and visible light communication system[J]. Small, 2019, 15(44): 1903599.

[5] Gao Y, Zhao C, Pu K, et al. Low-voltage-modulated perovskite/organic dual-band photodetectors for visible and near-infrared imaging[J]. Science Bulletin, 2022, 67(19): 1982-1990.

[6] Li C, Wang H, Wang F, et al. Ultrafast and broadband photodetectors based on a perovskite/organic bulk heterojunction for large-dynamic-range imaging[J]. Light: Science & Applications, 2020, 9(1): 31.

[7] He L, Hu G, Jiang J, et al. Highly Sensitive Tin‐Lead Perovskite Photodetectors with Over 450 Days Stability Enabled by Synergistic Engineering for Pulse Oximetry System[J]. Advanced Materials, 2023, 35(10): 2210016.

[8] LeCun Y, Bottou L, Bengio Y, et al. Gradient-based learning applied to document recognition[J]. Proceedings of the IEEE, 1998, 86(11): 2278-2324.

[9] Platt J. Sequential minimal optimization: A fast algorithm for training support vector machines[J]. 1998.

[10] Abeywickrama T, Cheema M A, Taniar D. K-nearest neighbors on road networks: a journey in

experimentation and in-memory implementation[J]. arXiv preprint arXiv:1601.01549, 2016.

[11] Hoerl A E, Kennard R W. Ridge regression: Biased estimation for nonorthogonal problems[J]. Technometrics, 1970, 12(1): 55-67.

[12] Freund Y, Schapire R E. A decision-theoretic generalization of on-line learning and an application to boosting[J]. Journal of computer and system sciences, 1997, 55(1): 119-139.

[13] Stanton J M. Galton, Pearson, and the peas: A brief history of linear regression for statistics instructors[J]. Journal of Statistics Education, 2001, 9(3).

[14] Geurts P, Ernst D, Wehenkel L. Extremely randomized trees[J]. Machine learning, 2006, 63: 3-42.

[15] Breiman L. Bagging predictors[J]. Machine learning, 1996, 24: 123-140.

[16] Quinlan J R. Induction of decision trees[J]. Machine learning, 1986, 1: 81-106.

[17] Breiman L. Random forests[J]. Machine learning, 2001, 45: 5-32.

[18] Friedman J H. Greedy function approximation: a gradient boosting machine[J]. Annals of statistics, 2001: 1189-1232.

[19] Lakshminarayanan B, Pritzel A, Blundell C. Simple and scalable predictive uncertainty estimation using deep ensembles[J]. Advances in neural information processing systems, 2017, 30.

[20] DeVries T, Taylor G W. Learning confidence for out-of-distribution detection in neural networks[J]. arXiv preprint arXiv:1802.04865, 2018.

[21] Gawlikowski J, Tassi C R N, Ali M, et al. A survey of uncertainty in deep neural networks[J]. Artificial Intelligence Review, 2023, 56(Suppl 1): 1513-1589.

[22] Clarke W L, Cox D, Gonder-Frederick L A, et al. Evaluating clinical accuracy of systems for self-monitoring of blood glucose[J]. Diabetes care, 1987, 10(5): 622-628.
